# Does patellofemoral osteoarthritis affect functional outcomes and survivorship after medial unicompartmental knee arthroplasty? A meta-analysis

**DOI:** 10.1186/s13018-020-02063-0

**Published:** 2020-12-04

**Authors:** Feifan Lu, Yan Yan, Weiguo Wang, Qidong Zhang, Wanshou Guo

**Affiliations:** 1 Peking University China-Japan Friendship School of Clinical Medicine, Peking University, Beijing, 100029 China; 2 Department of Orthopedic Surgery, Beijing Key Lab Immune-Mediated Inflammatory Diseases, Peking Union Medical School, China-Japan Friendship Hospital, Beijing, 100029 China

**Keywords:** Patellofemoral osteoarthritis, UKA, Meta-analysis

## Abstract

**Purpose:**

The argument that patellofemoral osteoarthritis (PFOA) is a contraindication to unicompartmental knee arthroplasty (UKA) remains to be resolved. The purpose of this study was to perform a meta-analysis to determine whether PFOA affects functional outcomes and survivorship after medial UKA.

**Methods:**

A literature search was performed in PubMed, Medline, Cochrane Library and Web of science to identify studies published in English (the last search was updated on June 1, 2020). The primary outcome measure was the Oxford Knee Score (OKS), whereas the secondary outcomes included range of motion (ROM) and the revision rate. Patients with patellofemoral joint narrowing or cartilage lesions as assessed intraoperatively or by radiography were assigned to the PFOA group.

**Results:**

A total of 8 studies involving 3504 patients and 3975 knees were included in this meta-analysis. No patients had a severe lateral patellar groove. The results showed that there was no significant difference in the OKS, revision rate and ROM between PFOA patients and patients without PFOA.

**Conclusions:**

No significant difference in functional outcomes and survivorship was found between patients with and without PFOA. Patients with PFOA assessed by radiographs or intraoperatively but without a lateral patellar groove should be considered candidates for medial UKA.

**Supplementary Information:**

The online version contains supplementary material available at 10.1186/s13018-020-02063-0.

## Introduction

Unicompartmental knee arthroplasty (UKA) is an effective method for the treatment of severe knee osteoarthritis and has received increasing attention. UKA can effectively relieve pain and improve function by surface replacement of the affected compartment and has the advantages of minimal trauma and a quick recovery. The long-term and medium-term functional results are satisfactory [1–3]. However, the contraindications of UKA are still controversial. Kozinn and Scott [4] suggested in 1989 that exposed bone in the patellofemoral joint should be regarded as a contraindication to UKA. In contrast, Goodfellow et al. [5] suggested that these contraindications can be ignored for mobile-bearing UKA. In anteromedial osteoarthritis, patellofemoral osteoarthritis (PFOA) is not a contraindication to UKA. In 2007, Beard et al. [5–7] proposed that PFOA should not be considered a contraindication to UKA as long as no groove is present in the lateral patella. Some recent studies suggest that the standard proposed by Kozinn and Scott was too strict and that UKA can be applied to more people with moderate patellofemoral joint degeneration [8].

The choice of surgery based on this limited evidence is questionable, and further study of the effect of the patellofemoral joint on functional outcome is needed. Therefore, the primary aim of this study was to determine whether PFOA affects functional outcomes after surgery, and the secondary aim of this study was to determine whether PFOA affects the survivorship of UKA by analysing the revision rate between the PFOA group and the group without PFOA. We hypothesised that PFOA without a lateral patellar groove does not affect the postoperative function and survival rate of UKA and was not contraindicated for UKA.

## Materials and methods

### Literature search strategy

We carried out a comprehensive literature retrieval by using the electronic databases PubMed, Medline, Cochrane Library and Web of Science to identify studies published in English (the last search was updated on June 1, 2020). The search strategy was based on the following keywords: (“UKA” OR “Unicompartmental knee arthroplasty”) AND (“PFOA” OR “patellofemoral osteoarthritis”). No other restrictions were placed on the search. Full text was obtained if the abstract was insufficient to allow us to include or exclude a study. Furthermore, the reference lists of all the related citations were examined to identify any initially omitted studies. All the literature searches were performed according to the Preferred Reporting Items for Systematic Reviews and Meta-Analyses (PRISMA) guidelines (Additional file).

### Inclusion and exclusion criteria

Two researchers screened the relevant investigations and identified eligible studies that met the following inclusion criteria: (1) examined patients with anteromedial osteoarthritis requiring primary medial UKA; (2) compared patients with patellofemoral osteoarthritis with patients without patellofemoral lesions; and (3) included at least one of the following outcomes: Oxford Knee Score (OKS), range of motion (ROM) and revision rate. Reviews, case reports, biochemical studies, letters and conference abstracts were excluded.

### Data extraction

Two researchers extracted all data independently according to the criteria described above. We developed a data extraction sheet including the year of publication, the first author’s name, study design, the mean age, male/female, UKA type, sample size, follow-up year, measurement time and PFOA assessment. Patients with patellofemoral joint narrowing or cartilage lesions as assessed intraoperatively or by radiography were assigned to the PFOA group. We classified a follow-up within 5 years as a short-term follow-up and a follow-up of 5–10 years as a medium-term follow-up. Any controversies of the data were discussed within our research team, and the authors reached a consensus on all items. The primary outcome measure was the OKS, whereas the secondary outcomes included ROM and revision rate. Some data, including range and median, were converted to mean and standard deviation (SD) for meta-analysis by the method provided by Hozo [9].

### Study quality assessment

The Newcastle-Ottawa Quality Assessment Scale (NOS) was used to assess the study quality [10]. The quality score of each study was based on three categories: selection (4 items, 1 point each), comparability (1 item, up to 2 points) and exposure/outcome (3 items, 1 point each). Each study scored from 0 points (worst) to 9 points (best) and scored 5 or less as low quality, whereas studies scoring 6 or higher were defined as high quality. The results of the study quality assessment are shown in Table 1.

**Table 1:** Study characteristics and patient demographic details

Author	Year	Design	Mean age(year)	Male/female	UKA type	Sample size (knees)		Follow-up year	NOS	Measurement time	Assessment of PFOA
						PFOA	N-PFOA				
Berend	2011	Comparative study	62.7	233/270	Mobile-bearing	74	564	1 to 7	6	Final follow-up	X-ray
Beard	2007	Cohort study	66	NC	Mobile-bearing	128	696	1 to 7	7	Final follow-up (mean 2 years)	By surgery
Pandit	2011	Cohort study	66.8	393/425	Mobile-bearing	158	842	1 to 10	7	Final follow-up (mean 5.6 years)	By surgery
Hamilton	2017	Cohort study	66	288/389	Mobile-bearing	190	615	5 to 17	7	10 years postoperatively	By surgery
Berger	2019	Cohort study	65	120/100	Fixed-bearing	57	183	2 to 5	7	Final follow-up (mean 4 years)	By surgery
Konan	2016	Cohort study	69	57/43	Mobile-bearing	52	48	8 to 13	7	Final follow-up (mean 10 years)	By surgery
Lim	2019	Cohort study	63	65/198	Fixed-bearing	41	222	8 to 12	7	10 years postoperatively	X-ray
Song	2016	Cohort study	64	3/96	Fixed-bearing	57	48	3 to 10	7	3 years postoperatively	By surgery

*PFOA* patellofemoral osteoarthritis, *N-PFOA* non-patellofemoral osteoarthritis, *NC* not clear, *NOS* Newcastle-Ottawa Quality Assessment Scale

### Statistical analysis

The mean differences (MD) with 95% confidence intervals (CIs) were calculated to analyse dichotomous outcomes. The relative risks (RRs) with 95% CIs were calculated to analyse dichotomous outcomes. Heterogeneity assumptions across studies were assessed by using the Q statistic with its *P* value and *I*^2^ statistic [11]. If *I*^2^ < 50% and *P* > 0.10, a fixed-effects model was used in the calculations; otherwise, a random-effects model was applied. In the evaluation of the primary outcome, a subgroup analysis was carried out according to the location of cartilage lesions of the patellofemoral joint (medial, lateral, trochlear and anywhere). Potential publication bias was assessed with a funnel plot. Sensitivity analysis was performed by omitting each study in turn to determine the impact on the heterogeneity test and to assess the stability of the overall results. All statistical analyses were conducted in Review Manager Software (RevMan version 5.3, the Cochrane Collaboration, Copenhagen, Denmark).

## Results

### Characteristics of the included studies

**Fig. 1:**
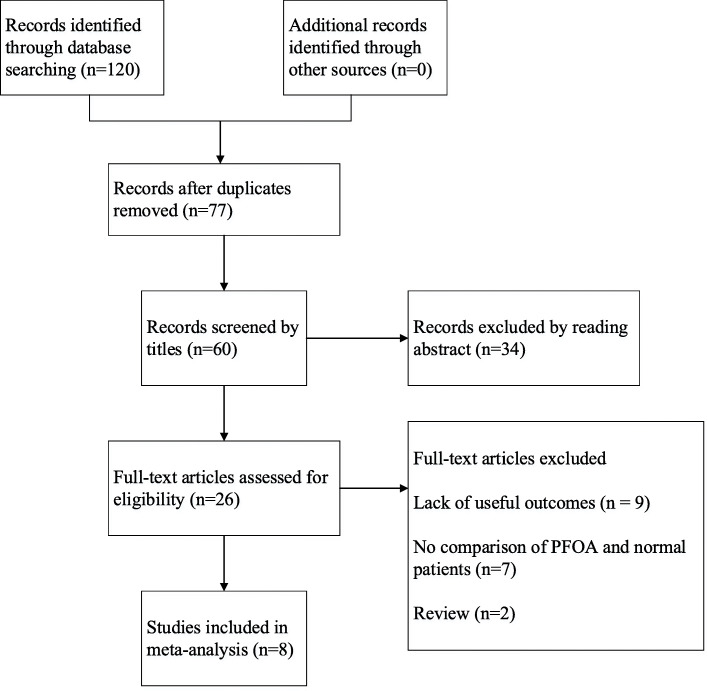
Flow diagram showing details of the literature search; PFOA, patellofemoral osteoarthritis

A total of 120 records were retrieved from the database, and 77 remained after eliminating duplicate documents. Then, 60 records were screened by titles, and 34 records were excluded after reviewing the abstracts. We reviewed the full text of the remaining 26 records and excluded 18 citations for reasons such as no comparison of patients with PFOA or not, lack of useful outcomes and reviews. Finally, we identified 8 studies [6, 12–18] in this meta-analysis (Fig. 1). A total of 3504 patients and 3975 knees were included in this meta-analysis. PFOA was assessed by preoperative radiography or intraoperative evaluation in all studies. No patients had a severe lateral patellar groove. More characteristics of the included studies are shown in Table 1.

### Meta-analysis

#### The primary outcome measurements

##### OKS

**Fig. 2:**
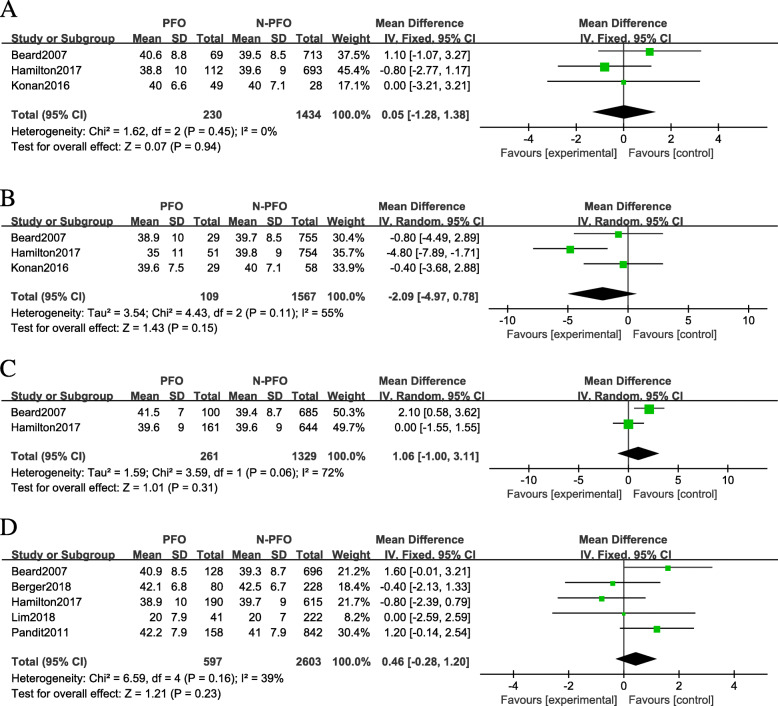
Forest plots of Oxford Knee Scores in the PFOA group and the non-PFOA group. **a** Medial. **b** Lateral. **c** Trochlear. **d** Anywhere in the PFJ. SD, standard deviation; CI, confidence interval

**Fig. 3:**
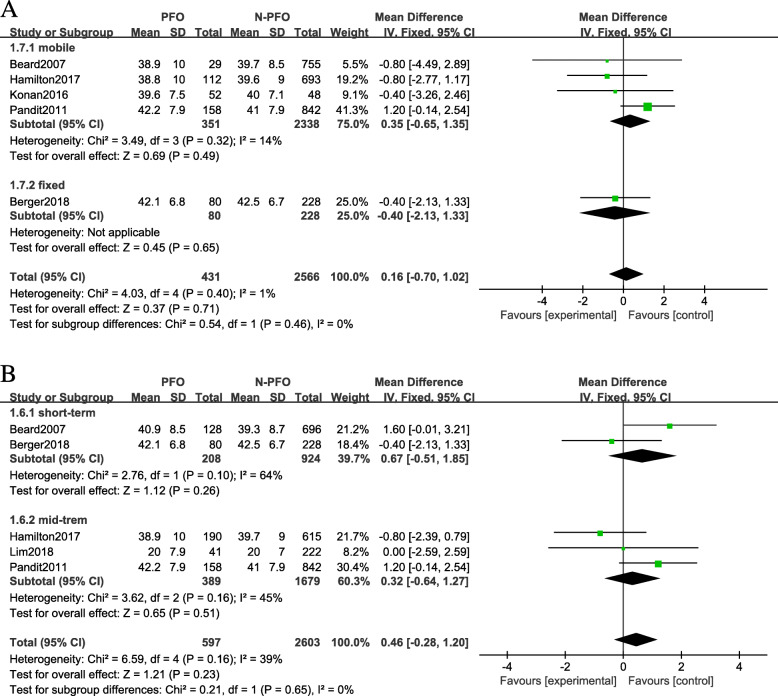
Forest plots of other outcomes in the PFOA group and the non-PFOA group. **a** Revision rate. **b** ROM

Seven studies that reported OKS results for a total of 3875 knees were included. On the basis of the location of cartilage lesions, we divided the results of OKS into four subgroups: (1) medial facet: (MD 0.05; 95% CI −1.28 to 1.38; *P* = 0.45; *I*^2^ = 0; fixed-effects model was used); (2) lateral facet: (MD −2.09; 95% CI −4.97 to 0.78; *P* = 0.11; *I*^2^ = 55%; random-effects model was used); (3) trochlear surface: (MD 1.06; 95% CI −1.00 to 3.11; *P* = 0.06; *I*^2^ = 72%; random-effects model was used); (4) anywhere in patellofemoral joint: (MD 0.46; 95% CI −0.28 to 1.20; *P* = 0.16; *I*^2^ = 39%; fixed-effects model was used, Fig. 2a–d). Subgroup analysis also showed that the UKA type (fixed-bearing and mobile-bearing) and measure time (short-term and mid-term) did not markedly affect the overall effect of the analysis (Fig. 3a-b).

#### The secondary outcome measurements

##### Revision rate

**Fig. 4:**
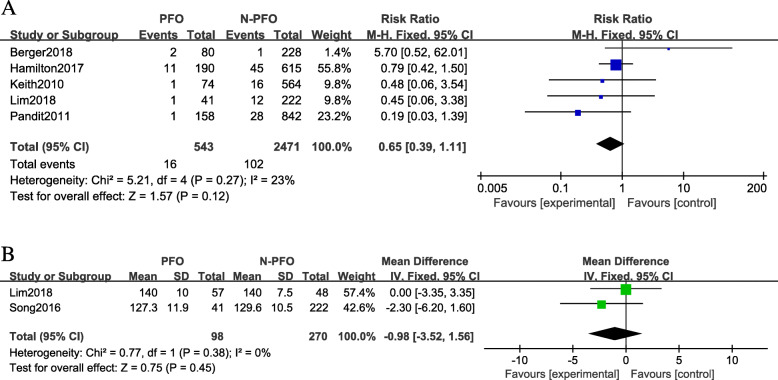
Subgroup analysis of forest plots of Oxford Knee Scores in the PFOA group and the non-PFOA group. **a** Mobile-bearing and fixed-bearing (**b**) measurements at the short-term follow-up and the mid-term follow-up

Five studies that included 3014 knees reported on revision rate were included. No significant difference was observed (RR 0.65; 95% CIs 0.39 to 1.1; *P* = 0.27; *I*^2^ = 23%) between the PFOA group and the control group (Fig. 4a).

##### ROM

Two studies with 368 knees reporting ROM outcomes were included. No significant difference was observed (MD −0.98; 95% CIs −3.52 to 1.56; *P* = 0.38; *I*^2^ = 0%) between the PFOA group and the normal group (Fig. 4b).

### Sensitivity analysis and publication bias analysis

**Fig. 5:**
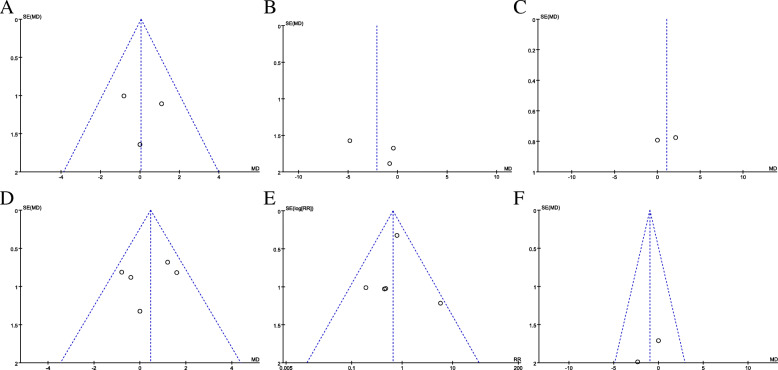
Funnel plot of the analysis. **a** OKS for medial facet lesions. **b** OKS for lateral facet lesions. **c** OKS for trochlear surface lesions. **d** OKS for lesions anywhere. **e** Revision rate. **f** ROM

Sensitivity analysis was conducted by omitting each study, in turn, to determine the effect on the heterogeneity test and evaluate the stability of the overall results. We found that the results in our sensitivity analysis were consistent with those in the non-sensitivity analysis, and the results indicated that our data were stable and credible. A funnel plot was generated to evaluate the publication bias of the literature. The results suggested that there was no evidence of publication bias in the meta-analyses (Fig. 5a–f).

## Discussion

To our knowledge, this is the first meta-analysis to directly compare whether patellofemoral osteoarthritis affects the clinical outcome of patients undergoing medial UKA. UKA is a popular method for the treatment of anteromedial osteoarthritis. However, whether the radiographic or clinical symptoms of PFOA are contraindications of UKA, it is still controversial [8, 19, 20]. Despite the lack of supporting evidence, many surgeons follow this intuitive advice. Therefore, the purpose of this meta-analysis is to make a relatively credible and comprehensive evaluation of whether patellofemoral osteoarthritis should be a contraindication of medial UKA.

The OKS can reflect the patient’s assessment of their knee-related health status and benefits of treatment [21, 22]. Therefore, we selected the OKS as our primary measurement to evaluate the functional outcome. Another reason was that of all eight studies included, seven articles included OKS as their results to reflect the outcome, so we can include more literature to increase the reliability of the results. Our meta-analysis showed no significant difference in OKSs between the PFOA group and the control group. Our subsequent subgroup analysis showed that there was no significant difference in OKS between patients with patellofemoral joint lesions and those with normal patellofemoral joints regardless of the lesion location, whether medial, lateral, trochlear or anywhere of the PFJ. This suggests that patellofemoral osteoarthritis may not be a contraindication of medial UKA. There was an argument that although the state of the patellofemoral joint does not affect the outcome of patients undergoing UKA, lateral patellofemoral osteoarthritis should still be regarded as a contraindication of UKA [23]. Our study found that moderate lateral PFOA had no significant effect on the mid-term follow-up of patients undergoing UKA. Some studies that were not included in the study due to different measurement data also suggested that PFOA might not significantly change the functional score after UKA [24, 25]. No included patients included had a lateral patellar groove, and whether patients with severe PFOA and a lateral patellar groove are candidates for UKA requires more supporting evidence. Some studies suggest that a lateral PF joint groove or subluxation may affect the survival rate of UKA [26, 27]. However, most scholars still regard a lateral patellar groove as a contraindication, and further studies may be needed to confirm this view.

In the five articles included, the revision rate of the PFOA group was lower than that of the control group, but the difference was not statistically significant (RR 0.65; 95% CIs 0.39 to 1.1). Moreover, the reason for UKA’s failure in the renovation is not due to PFOA’s progress [14]. This suggested that the progression of PFOA is not the main cause of UKA failure, and patients with patellofemoral joint lesions receiving medial UKA do not have an increased risk of UKA failure.

One of the main reasons why patellofemoral joint lesions do not affect the postoperative function or survival of UKA is that most people are asymptomatic. Among 34- to 55-year-olds, asymptomatic radiologic evidence has been reported to show a 30% incidence of PFJ osteoarthritis, with autopsy studies showing that almost all elderly people who have not reported knee pain have PFOA [28, 29]. Noble and Hamblen reported a 79% incidence rate of PFOA among 100 randomly selected corpses aged >65 years [30]. Therefore, in most patients who need knee arthroplasty, including those with painful medial OA, PFOA may be asymptomatic, so it will not affect the prognosis of UKA. Because the presence of knee pain before UKA has nothing to do with the state of PFOA, it may be related to the medial OA, and it will disappear after UKA. In addition, if a patient has an abnormal patella tracking, a mobile-design UKA may restore the normal patella track to restore the alignment of the limbs to restore normal function and minimise the risk of complications [14].

This study still has some limitations. First, the follow-up time of most of the literature included was short, and there was no uniform follow-up time. Therefore, we divided the follow-up time into groups, and there was no difference between the short-term and medium-term follow-up groups. Second, because of the different clinical outcomes adopted by the included literature, there are few outcome indicators finally included in the analysis, and some of them contain only a few studies. Third, despite subgroup analysis, heterogeneity is inevitable, and we use the random effect model to minimise the impact of heterogeneity. Fourth, due to the inclusion of English literature only, publication bias is inevitable. Finally, the total number of articles included was still small and unable to explain all the results, which may need further research to confirm.

## Conclusion

To our knowledge, this is the first meta-analysis to directly compare whether patellofemoral osteoarthritis affects the clinical outcome of patients undergoing UKA. The results showed that there was no significant difference in OKS, revision rate or ROM between patients with PFOA and patients without PFOA. On the basis of these findings, we conclude that patients with PFOA assessed intraoperatively or by radiography but without a lateral patellar groove should be considered candidates for medial UKA.

## Supplementary Information

**Additional file 1.**

## Data Availability

Not applicable.

## Abbreviations

UKA: Unicompartmental knee arthroplasty
CIs: Confidence intervals
MD: Mean difference
RR: Relative ratio
PFOA: Patellofemoral osteoarthritis
OKS: Oxford Knee Score
ROM: Range of motion
